# Macropinocytosis: New Insights Into Its Underappreciated Role in Innate Immune Cell Surveillance

**DOI:** 10.3389/fimmu.2018.02286

**Published:** 2018-10-02

**Authors:** Johnathan Canton

**Affiliations:** Immunobiology Laboratory, The Francis Crick Institute, London, United Kingdom

**Keywords:** macropinocytosis, macropinosome, PRRs, pattern recognition receptors (PRRs), antigen presentation, macrophages, dendritic cells, Nod2 signaling adaptor protein

## Abstract

Macropinocytosis has received increasing attention in recent years for its various roles in nutrient acquisition, immune surveillance, and virus and cancer pathologies. In most cases macropinocytosis is initiated by the sudden increase in an external stimulus such as a growth factor. This “induced” form of macropinocytosis has been the subject of much of the work addressing its mechanism and function over the years. An alternative, “constitutive” form of macropinocytosis restricted to primary innate immune cells also exists, although its mechanism has remained severely understudied. This mini-review focuses on the very recent advances that have shed new light on the initiation, formation and functional relevance of constitutive macropinocytosis in primary innate immune cells. An emphasis is placed on how this new understanding of constitutive macropinocytosis is helping to define the sentinel function of innate immune cells including polarized macrophages and dendritic cells.

## Introduction

The ability of eukaryotic cells to internalize extracellular material was first recognized at the turn of the nineteenth century by Ilya Metchnikoff (1). Metchnikoff documented the internalization of large particulate structures by amoeboid cells in starfish larva and referred to the process as phagocytosis, or “cell eating.” Almost half a century later, in 1931, Warren H. Lewis observed the uptake of extracellular fluid by cells *in vitro* and coined the term pinocytosis, or “cell drinking” (2). In the time since it was first observed, pinocytosis has been recognized to represent a broad array of cellular internalization, or endocytic, pathways including clathrin-mediated endocytosis, caveolae-dependent uptake and the CLIC/GEEC pathway. These pathways represent entire fields of study and continue to be actively investigated. One pinocytic pathway, however, has received far less attention—macropinocytosis.

Macropinocytosis is initiated by the actin-driven extension of plasma membrane ruffles. Occasionally, membrane ruffles form a cup-like structure or nascent macropinosome that seals at its distal tips to form a relatively large (>250 nm), phase-bright endosome termed a macropinosome. Subsequent membrane fusion and fission interactions with various organelles, primarily components of the endocytic pathway, result in the formation of a mature, acidic, and often tubular structure termed a macropino-lysosome. This series of events has been well-documented for growth factor-induced macropinocytosis in non-myeloid cell types and has been reviewed extensively elsewhere (3–5). Somewhat surprisingly though, it has been tacitly assumed that these mechanistic insights transfer to cells of the innate immune system.

More recently, the constitutive form of macropinocytosis in innate immune cells, specifically human monocyte-derived macrophages (hMDMs) and human monocyte-derived dendritic cells (hMDCs), has in fact been found to be mechanistically different. Constitutive macropinocytosis, for example, requires the presence of extracellular calcium, is less sensitive to perturbations in intracellular/cytosolic pH and forms morphologically distinct macropinosomes [for a detailed comparison of growth-factor induced and constitutive macropinocytosis, please see reference (6)]. Moreover, constitutive macropinocytosis appears to be restricted to primary innate immune cells, most often immature dendritic cells and macrophages, and has been observed in these cell types both *in vitro* and *in vivo* (7–9). The rate at which constitutive macropinocytosis occurs is staggering. Indeed, in their pioneering work, Steinman et al. observed macrophages internalizing their entire cell surface every 33 min (10). Given the significant energy cost of undergoing such an astoundingly active mode of endocytosis, the evolutionary conservation of constitutive macropinocytosis attests to its functional significance. Nevertheless, it remains severely understudied in dendritic cells and even more so in macrophages.

The lack of understanding of constitutive macropinocytosis in primary innate immune cells is largely a result of the notorious difficulty in both the culture and genetic manipulation of primary immune cells. Fortunately, recent advances in our understanding of innate immune cell ontogeny and culture as well as improved systems for the genetic manipulation of primary cells, such as the CRISPR technology, have allowed for a renewal of interest in this phenomenon. This mini-review will place its focus on the constitutive form of macropinocytosis in primary polarized macrophages and dendritic cells. Recent revelations in the initiation, mechanism, and functional relevance will be discussed. Trends in the field will be highlighted and a new perspective on how constitutive macropinocytosis is essential to the functioning of macrophages and dendritic cells in homeostasis, immune surveillance and the initiation of an adaptive immune response will be proposed.

## The mechanics of constitutive macropinocytosis

**Figure 1:**
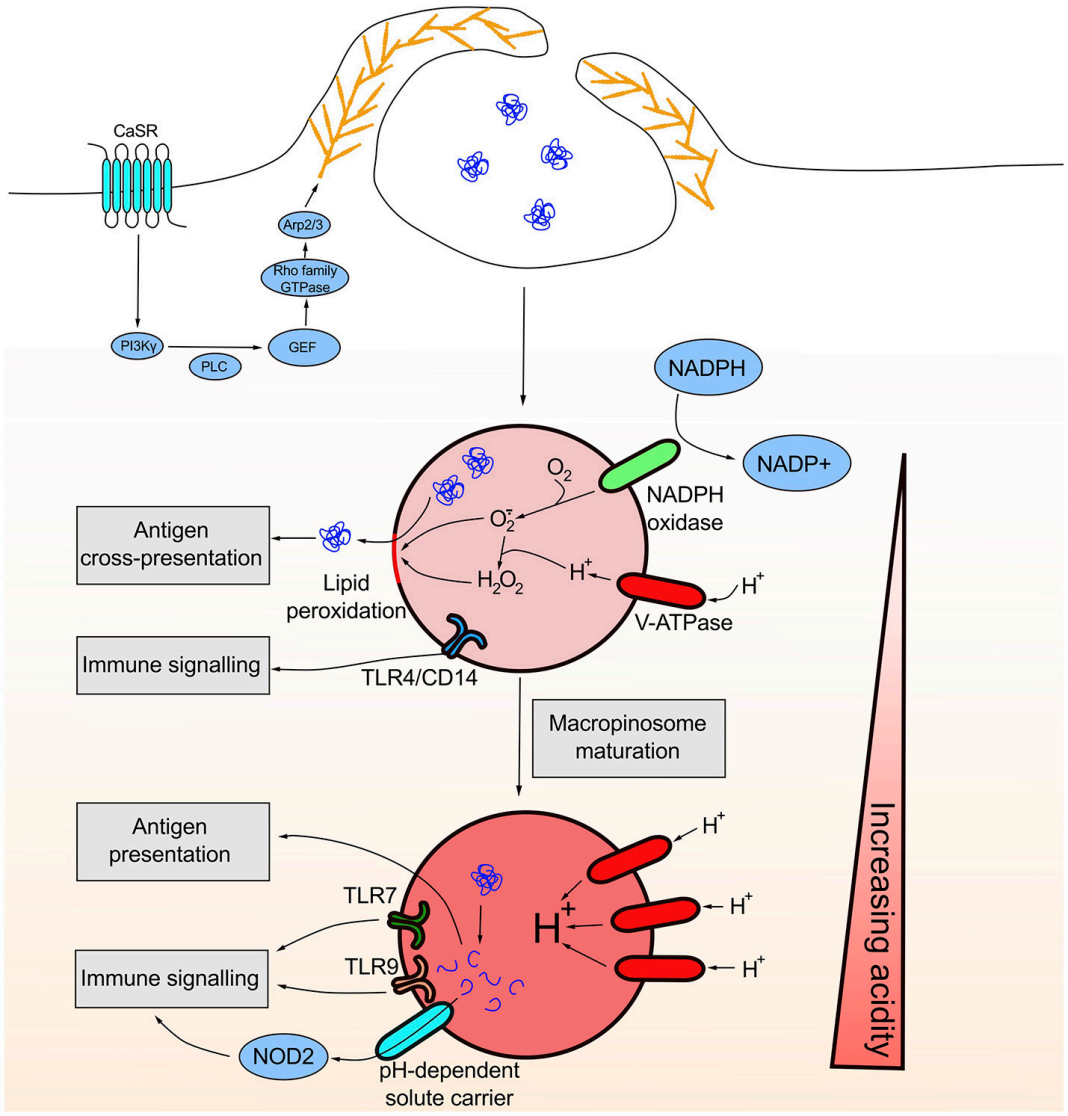
Constitutive macropinocytosis is initiated by the sensing of extracellular calcium by the G protein-coupled receptor CaSR. CaSR ligation initiates a signaling cascade ultimately resulting in the elaboration of an Arp2/3 dependent branched actin network at the plasma membrane that drives constitutive ruffling. Early macropinosomes are less acidic due to limited contact with late endosomes/lysosomes and likely NADPH oxidase activity. The generation of reactive oxygen species damages the integrity of the macropinosome membrane through lipid peroxidation thus allowing for the release of antigen into the cytosol necessary for subsequent cross-presentation. As the macropinosome matures, the lumen becomes increasingly acidic allowing for the activation of protease and lipases with acidic pH optima. This degradative environment facilitates the generation of peptides that can bind MHCII for presentation. Similarly, the degradative environment can liberate NOD1/2 ligands that can be transported in a pH-dependent manner across the macropinosomal membrane into the cytosol where they can bind cytosolic PRRs. Luminal ligands for membrane bound PRRs such as TLR4, TLR7 and TLR9 can also bind their respective receptors once internalized.

Innate immune cells, like macrophages and immature dendritic cells, continuously elaborate dynamic membrane protrusions in a process referred to as membrane ruffling. This process is essential to both the housekeeping and immune functioning of these cells as it facilitates the capture of phagocytic targets thereby dramatically improving the efficiency with which they clear both potential pathogens and metabolic debris (11). Membrane ruffling is also the first step to forming a macropinosome (Figure 1) (12). This can be initiated by the sudden increase in concentration of an inducing stimulus such as a growth factor or microbe associated molecular pattern (MAMP) (8, 12). This induced form of macropinocytosis is not unique to macrophages and dendritic cells and has been reviewed elsewhere (5, 13). However, the constitutive ruffles mentioned above also occasionally seal at their distal tips to form macropinosomes. This constitutive form of macropinocytosis is unique to innate immune cells, with the notable exception of oncogene-transformed malignant cells (14).

The question of whether or not the constitutive ruffling of phagocytes was initiated and maintained by an ever present external stimulus or was the result of a cell intrinsic constitutively active driver of membrane protrusions had remained a mystery until very recently. In a study comparing growth factor induced and constitutive macropinocytosis in primary human monocyte-derived macrophages and primary human monocyte-derived dendritic cells, it was shown that extracellular calcium is the driver of constitutive macropinocytosis (6, 9 macropinocytosis or phagocytosis). Extracellular calcium is sensed through the G protein-coupled receptor (GPCR) calcium sensing receptor (CaSR), which upon ligation results in the transient generation of key lipid mediators on the cytosolic leaflet of the plasma membrane—notably phosphatidic acid (PtdOH) and phosphatidylinositol 3,4,5-*tris*phosphate [PtdIns(3,4,5)P_3_]. PtdOH and PtdIns(3,4,5)P_3_ coordinate the recruitment and activation of actin nucleators at the plasma membrane in several converging steps. Firstly, several guanine nucleotide exchange factors (GEFs), such as DOCK2 and TIAM1 (15, 16), harbor polybasic domains that are electrostatically attracted to the negatively charged head groups of the phosphoinositide intermediates generated at the plasma membrane. GEFs in turn activate small GTPases, such as Rac1/2, that drive actin polymerization via the activation of downstream actin nucleation promoting complexes, such as the SCAR/WAVE complex and WASp (17–19). Another lipid mediator, phosphatidylinositol 4,5-*bis*phosphate [PtdIns(4,5)P_2_] is also transiently generated at sites of ruffle formation through the recruitment of type I phosphatidylinositol phosphate kinase by PtdOH (15, 20, 21). PtdIns(4,5)P_2_ can be further converted into either PtdIns(3,4,5)P_3_ through the action of phosphatidylinositol 3-kinase or hydrolyzed into diacylglycerol (DAG) and inositol 3,4,5-*tris*phosphate (IP_3_) by phospholipases (12, 22, 23). DAG is then converted into PtdOH by diacylglycerol kinase (DGK) thereby bolstering the aforementioned activation of actin nucleation promoting complexes. Ultimately, these events result in the formation of an Arp2/3-dependent branched actin network at the plasma membrane driving dynamic protrusions from the dorsal surface of the cell (Figure 1). Importantly, in the absence of extracellular calcium neither PtdIns(3,4,5)P_3_ nor PtdOH is generated at the plasma membrane in phagocytes outlining the importance of this signal in the maintenance of constitutive ruffle formation (6).

A membrane ruffle, however, is not a macropinosome. Only a small fraction of the membrane ruffles generated by phagocytes seal to become macropinosomes. Whether or not there are specialized ruffles that are destined to become macropinosomes has been the subject of speculation for quite some time. Joel Swanson and colleagues have shown that in addition to the numerous linear protrusions from the plasma membrane, specialized circular ruffles enriched in PtdIns(3,4,5)P_3_ were likely to seal into macropinsomes (23), but the form of macropinocytosis being studied was growth factor induced and not constitutive macropinocytosis. This is also complicated by the observation that in primary human macrophages and dendritic cells, which ruffle constitutively, PtdIns(3,4,5)P_3_ appears to be enriched in numerous ruffles at the plasma membrane, some of which do not seal to form macropinosomes (6, 9). Moreover, it was recently shown that the sequential hydrolysis of PtdIns(3,4,5)P_3_ into phosphatidylinositol 3,4-*bis*phosphate and ultimately into phosphatidylinositol 3-phosphate is required for the closure of ruffles into macropinosomes (22), but exactly how that promotes closure is still unknown. However, new techniques allowing for greater spatial and temporal resolution of individual ruffles, such as lattice light sheet microscopy, are now being applied to the study of macropinocytosis in *Dictyostelium* (24) and could potentially allow for the visualization of individual nascent macropinosomes in mammalian phagocytes. Indeed, there is much to be learnt about the mechanics of macropinosome formation.

## The function of constitutive macropinocytosis: the evidence for antigen presentation

Growth factor induced macropinocytosis has a well-established role in the acquisition of extracellular solutes/nutrients in support of cell growth and metabolism (13). The functions of constitutive macropinocytosis on the other hand are significantly less clear. Given its relative specificity to macrophages and more notably immature dendritic cells, a role in the acquisition of antigen for subsequent processing and presentation to the adaptive arm of the immune system has been proposed (Figure 1) (7, 25–31). The notion that the primary function of constitutive macropinocytosis is to serve as a major route for the internalization of exogenous antigen however stands on strikingly shaky ground and deserves further consideration.

Antigen-presenting cells use a broad range of endocytic pathways for the internalization of exogenous antigen for processing and presentation (32). Therefore, linking constitutive macropinocytosis to antigen presentation requires methods for the specific manipulation of this mode of endocytosis. To date, an incomplete understanding of the mechanism of constitutive macropinocytosis along with its similarity to other modes of endocytosis, has left us with a dearth of techniques for the specific manipulation of macropinocytosis *in vitro* and even more so *in vivo*. Indeed, many of the pharmacological inhibitors of macropinocytosis are notoriously non-specific. For example, amiloride derivatives, one of the most frequently used inhibitors of macropinocytosis (13, 33–35), are in fact promiscuous inhibitors of Na^+^/H^+^ exchangers (36), Na^+^ channels (37) and Na^+^/Ca^2+^ exchangers (38). The inhibitory effect of amiloride derivatives is a result of the dysregulation of submembranous pH due to impaired Na^+^/H^+^ exchange at sites of macropinocytosis (34). Importantly, this effect is neither specific to macropinocytosis nor to cell types capable of performing macropinocytosis. Yet, this inhibitor has been used to probe the effect of macropinocytosis on antigen presentation even *in vivo* (7) where it almost certainly has non-specific effects on a very wide range of cell types, including other immune cell types (39). Therefore, the effect of amiloride derivatives on macropinocytosis is incidental and cannot be used alone as a specific inhibitor of macropinocytosis. Moreover, their effect on antigen presentation *in vivo* need not be linked to macropinocytosis as Na^+^/H^+^ exchangers have been linked to a wide range of immune cell functions including cell migration, apoptosis, differentiation, and even proliferation (40). It is also worth noting that, unlike growth factor induced macropinocytosis, the constitutive form of macropinocytosis is only marginally sensitive to amiloride derivatives (6). Other pharmacological agents including rottlerin (30, 41), rapamycin (25), and sanglifehrin A (26) have been used to study antigen uptake by macropinocytosis both *in vitro* and *in vivo* but are similarly non-specific and only limited conclusions can be drawn from such studies.

A more specific way to study macropinocytosis employs tagged fluid-phase tracers often in conjunction with pharmacological inhibition. An important consideration is the hydrodynamic radius of the fluid phase tracer, which must be sufficiently large so as to not be taken up via other modes of endocytosis. For example, soluble, fluorescently-tagged 70 kDa dextran is large enough to be selectively taken up by macropinocytosis, but not other smaller forms of endocytosis such as clathrin-mediated endocytosis or caveolae (42). Somewhat surprisingly, the majority of the studies linking macropinocytosis to antigen presentation have used either fluid-phase tracers that can be taken up by various forms of endocytosis or fluorescent dextran molecules of unknown size (7, 25, 28, 29, 31, 43, 44). For example, fluorescently tagged ovalbumin is frequently used to study antigen uptake by macropinocytosis (27, 31, 43), yet it is known that ovalbumin uptake by myeloid cells occurs predominantly via the mannose receptor in clathrin-coated pits (45, 46).

Clearly, the contribution of constitutive macropinocytosis to the uptake of antigen and its subsequent presentation could benefit from a more detailed understanding of its mechanism and therefore a more targeted assessment of tis role. Recent findings are bringing us closer to that goal. A selective agonist of the CaSR, NPS2143, inhibits constitutive macropinocytosis in primary human macrophages and dendritic cells without affecting clathrin-dependent endocytosis, growth factor induced macropinocytosis or phagocytosis (6, 9). NPS2143 represents a potentially powerful tool for assessing antigen uptake via macropinocytosis *in vitro*; however, its use *in vivo* is limited due to expression of CaSR by other cell types. Nevertheless, the myeloid-specific deletion of CaSR *in vivo* could bring us closer to understanding the role of constitutive macropinocytosis in antigen presentation.

## Are there other functions for constitutive macropinocytosis?

Given the enormous volume of extracellular fluid that primary macrophages and dendritic cells turn over via constitutive macropinocytosis, it is likely that antigen, as discussed above, can enter for subsequent processing and presentation (Figure 1). Bulk extracellular fluid uptake however is not a particularly efficient means of internalizing antigen. Indeed, mathematical modeling has shown that receptor-dependent uptake, as opposed to fluid phase bulk uptake, requires 1,000-fold less antigen for efficient internalization and subsequent presentation (47). Moreover, receptor-dependent uptake provides a certain degree of selectivity for self vs. non-self cargo and allows for the sorting of antigen to an intracellular compartment that is competent for antigen presentation (32, 48). It is entirely unclear whether mechanisms for the concentration of antigen exist or if the differential sorting of macropinocytic cargo occurs after macropinosome formation. In addition, the observation that other antigen presenting cells like B cells, mature dendritic cells and inflammatory macrophages, all of which do not perform macropinocytosis (44, 49), are still capable of internalizing and presenting antigen (32, 50, 51) begs the question of whether constitutive macropinocytosis may serve other functions.

We have recently proposed a novel surveillance function for constitutive macropinocytosis in macrophages and immature dendritic cells. Both macrophages and dendritic cells express a broad array of pattern recognition receptors (PRRs) for the detection of non-self and/or altered-self material in the extracellular milieu. PRRs are essential to the sentinel function of myeloid cells in that they allow them to respond to potential pathogens or damaged cells through the detection of MAMPs or damage associated molecular patterns (DAMPs), respectively. Myeloid cells respond by initiating gene transcription and translation pathways that result in both metastable changes to myeloid cell function and the production of soluble signals that can propagate responses to neighboring cells [reviewed in (52–56)]. Although many PRRs are located on the exofacial leaflet of the plasma membrane, others are located in intracellular compartments (Figure 1) (57). Ligands for intracellular PRRs can be delivered via receptor-mediated endocytosis and phagocytosis [reviewed in (58)]. However, under circumstances where neither receptor-mediated endocytosis nor phagocytosis apply, macropincytosis serves as an ideal means for delivering ligands to intracellular PRRs. This is most convincingly revealed through the study of outer membrane vesicles (OMVs) that are shed by microbes during infection. OMVs, which can have a diameter larger than 300 nm (59), are shed by Gram-negative bacteria and have been implicated in the establishment of a replicative niche by pathogenic species (60). Importantly, OMVs can travel to sites that are distant from the initial site of infection and activate intracellular PRRs such as NOD1 (61–64). Interestingly, biophysical studies of receptor-mediated endocytosis have determined that the size optimum for the uptake of spherical particles is around 25–30 nm, placing the large majority of OMVs squarely outside the optimal range for uptake via mechanisms such as clathrin-mediated endocytosis (65–68). Likewise, the size optimum for the uptake of particles by phagocytosis is between 2 and 3 μm with efficiency decreasing dramatically when particles are below 1 μm, rendering phagocytosis of OMVs unlikely (69). Macropinosomes vary dramatically in size and can be anywhere from 250 nm to 5 μm in size, which is favorable for the uptake of OMVs (3, 4). It is conceivable then that macropinocytosis can facilitate the capture of OMVs and the delivery of intracellular PRR ligands present on or within OMVs to their respective receptors. In line with this, we have shown that macropinosomes are competent in the delivery of the PRR NOD1/2 ligands to the cytosol for sensing (6), most likely via pH-dependent solute carriers present on the membrane of the macropinosomes (Figure 1) (70–72). The contribution of constitutive macropinocytosis to the delivery of ligand to intracellular PRRs is sufficiently large that acutely turning off constitutive macropinocytosis completely ablates NOD1 signaling in macrophages (6).

The above observations notwithstanding, very few studies have begun to explore the uptake of OMVs, let alone other material shed and/or secreted by potential pathogens, by constitutive macropinocytosis (73, 74). After sealing, macropinosomes quickly acidify (75). Acidification is largely due to the delivery and accumulation of the vacuolar H^+^-ATPase (V-ATPase) on the macropinsomal membrane through membrane fusion and fission events with components of the late endocytic and lysosomal compartments (76). The rate of acidification is also likely to be influenced by several additional factors including the buffering power of the luminal contents, the rate of proton leakage, consumption of protons by reactive oxygen species (ROS) generated by the NADPH oxidase (Figure 1), and the permeability of the macropinosomal membrane to counterions (77–79). Luminal acidification activates various hydrolases, lipases, and proteases with acidic pH optima (5). The degradative environment serves as an ideal compartment for the unmasking and ultimate pH-dependent delivery of PRR ligands to the cytosol. Similarly, several transmembrane PRRs either require an acidic compartment for optimal binding to their ligands, as is the case for TLR3 (80), change their downstream signaling once internalized, as is the case for TLR4 (81), or are predominantly located in an endolysosomal compartment, as is the case for TLR7 and TLR9 (Figure 1) (82). Indeed, new studies are beginning to show that macropinosomes do serve as specialized signaling platforms for various intracellular TLRs (83).

A clear role for macropinocytosis in immune surveillance, not just antigen presentation, begins to emerge. Of interest is the tunability of constitutive macropinocytosis by myeloid cells. Homeostatic macrophages and anti-inflammatory macrophages, for example, perform constitutive macropinocytosis. Inflammatory macrophages do not undergo constitutive macropinocytosis (9). As such, macrophages can control under what conditions PRR ligands are delivered to their intracellular receptors, and whether certain PRRs encounter ligand at the plasma membrane or in an intracellular compartment. This becomes particularly relevant at sites of continuous contact with non-self material such as in the lung or gut. Interestingly, OMVs produced by microbes in the gut are internalized by gut-resident macrophages and dendritic cells and have an immunomodulatory effect (84, 85). Dendritic cells can also control their macropinocytic activity as they diminish their capacity for macropinocytosis upon maturation (44, 49, 86). Although this may largely represent a shift from antigen capture to the maintenance of MHC complexes on a quiescent plasma membrane, it too may impact the signaling capacity of PRRs both in intracellular compartments and at the plasma membrane. Intriguingly, through unclear mechanisms, extracellular calcium is elevated at sites of tissue injury and infection (87, 88) and myeloid cells have been shown to sense these changes via the CaSR (88). Given the dose-dependent nature of calcium-dependent constitutive macropinocytosis (6), local increases in extracellular calcium at sites of tissue injury and infection also represent an opportunity for enhancing macropinocytic activity and therefore the delivery of PRRs to intracellular compartments. Constitutive macropinocytosis then represents not only a means for antigen capture, but a tunable and efficient mechanism for the delivery of ligand to PRRs.

## Conclusion

Innate immune cells are considered sentinels, uniquely endowed with an armory of PRRs that allow them to sense and distinguish between self, altered self, commensal non-self and pathogenic non-self material. The mechanics of sensing through PRRs is far more nuanced than previously appreciated and it is increasingly acknowledged that the subcellular compartmentalization of signaling platforms results in distinct responses. The enormous energy cost incurred by macrophages and dendritic cells in the performance of constitutive macropinocytosis was previously explained as a means of improving antigen capture. Indeed antigen can be captured by bulk fluid uptake although, as discussed above, it is not a particularly efficient means of doing so. A new function for constitutive macropinocytosis is emerging in the delivery of ligands to their respective intracellular PRRs. As such, constitutive macropinocytosis, which is unique to dendritic cells and macrophages, facilitates a defining feature of this subset of cells—immune surveillance. A closer look at how macropinosomes serve as signaling platforms and how they facilitate the delivery of ligands to both membrane bound and cytosolic PRRs is warranted. An improved knowledge of constitutive macropinocytosisis is therefore essential as it has clear implications in the regulation of immunity.

## Author contributions

The author confirms being the sole contributor of this work and has approved it for publication.

### Conflict of interest statement

The author declares that the research was conducted in the absence of any commercial or financial relationships that could be construed as a potential conflict of interest. The reviewer GS and handling Editor declared their shared affiliation.
